# Genetic interference exerted by *Salmonella-*delivered CRISPR/Cas9 significantly reduces the pathological burden caused by Marek’s disease virus in chickens

**DOI:** 10.1186/s13567-021-00995-x

**Published:** 2021-09-30

**Authors:** Amal Senevirathne, Chamith Hewawaduge, John Hwa Lee

**Affiliations:** College of Veterinary Medicine, Chonbuk National University, Iksan Campus, 54596 Iksan, Republic of Korea

**Keywords:** *Salmonella*, CRISPR/Cas9, virus-induced cancer, qRT–PCR, genomic interference

## Abstract

Efficient in vivo delivery of a CRISPR/Cas9 plasmid is of paramount importance for effective therapy. Here, we investigated the usability of *Salmonella* as a plasmid carrier for in vivo therapy against virus-induced cancer using Marek’s disease virus (MDV) as a model for study in chickens. A green fluorescent protein-expressing CRISPR/Cas9 plasmid encoding the virulence gene *pp38* was constructed against Marek’s disease virus. Therapeutic plasmids were transformed into *Salmonella* carrying *lon* and *sifA* gene deletions. The animals in 5 groups were intraperitoneally inoculated with phosphate-buffered saline, vector control, or *Salmonella* before or after MDV infection, or left uninfected as a naïve control. Therapeutic effectiveness was evaluated by observing disease outcomes and the viral copy number in peripheral blood mononuclear cells. The efficacy of plasmid delivery by *Salmonella* was 13 ± 1.7% in the spleen and 8.0 ± 1.8% in the liver on the 6^th^ day post-infection. The *Salmonella*-treated groups showed significant resistance to MDV infection. The maximum effect was observed in the group treated with *Salmonella* before MDV infection. None of the chickens fully recovered; however, the results suggested that timely delivery of *Salmonella* could be effective for in vivo CRISPR/Cas9-mediated genetic interference against highly pathogenic MDV. The use of *Salmonella* in CRISPR systems provides a simpler and more efficient platform for in vivo therapy with CRISPR than the use of conventional in vivo gene delivery methods and warrants further development.

**Supplementary Information:**

The online version contains supplementary material available at 10.1186/s13567-021-00995-x.

## Introduction

The clustered regularly interspaced palindromic repeats and associated protein 9 (CRISPR/Cas9) system has been widely recognized as the most robust genome editing platform available in basic biomedical sciences [1–3]. Efficient delivery of the CRISPR/Cas9 system into target cells is essential for achieving the expected outcome. Although viral vectors are widely used [4] for delivery of the CRISPR/Cas9 system in vitro and in vivo, their fundamental shortcomings, such as the risk of carcinogenesis, limited insertion size, immune response induction, difficulty in large-scale production, genomic manipulation issues, and narrow tissue tropism, impose considerable limitations [5, 6]. Alternative nonviral delivery platforms, such as lipid- or polymer-based nanoparticle [7] and exosomal [8, 9] strategies, have also been attempted; however, these strategies are noninvasive, and their efficacy under in vivo conditions is very low. In the present context, despite its tremendous potential, the utilization of bacterial species for CRISPR in vivo delivery has not been sufficiently investigated. Bacterial species such as attenuated *Salmonella* Typhimurium (ST) have demonstrated commendable potential in in vivo therapy due to their natural tropism for the host lymphoid system and antigen-presenting cells. These species are highly efficient in invading both phagocytic cells and nonphagocytic cells and preferentially populate hypoxic tumours [10, 11]. Their remarkable survival in immunocompetent hosts is also essential for the successful distribution and delivery of CRISPR plasmids into target host tissues [12]. In contrast to viral vectors, *Salmonella* could offer many more advantages in regard to payload capacity, scalability, and manoeuvrability [13].

Among all human cancers, more than 15% have an incidence connected with viral infections [14, 15]. Most virus-induced diseases in humans still do not have effective treatment strategies, yet the relationship between viruses and cancer is becoming increasingly apparent. Marek’s disease is a lymphoproliferative disorder in avian species caused by Gallid herpesvirus 2 (GaHV-2) [16]. Marek’s disease virus (MDV) is highly contagious, and infection results in severe malignancies in the spleen, liver, and other peripheral organs following the expression of viral genes [16, 17]. Immunization of chickens is the only successful strategy to control MDV [18]; however, vaccination does not provide sterilizing immunity [19]. Hence, MDV field strains can still replicate in and disseminate via immunized birds. Furthermore, virus-host adaptation may lead to more virulent strains that can overcome the immunization barrier. Therefore, vaccines that operate beyond the conventional concept of immune induction could be highly important. It is worth exploring novel strategies, such as CRISPR/Cas9-mediated genomic interference techniques, to assess whether such a system is capable of arresting viral infection. Experiments conducted by Hagag et al. revealed that the use of multiple genomic targets can completely inhibit MDV infection under in vitro conditions [20, 21]. Transgenic chickens constitutively expressing ICP4-targeting sgRNA and the Cas9 protein exhibit significant protection against MDV infection [21]. However, the majority of poultry birds are nontransgenic; therefore, an efficient platform for CRISPR therapy is essential for developing this system into a viable preventive strategy. Under field conditions, efficient delivery and plasmid release in target sites demarcate a key success determinant. In this study, we used *Salmonella* to deliver a therapeutic CRISPR/Cas9 plasmid directly into the sites of early infection, such as macrophages in the lung epithelium, as a remedy for MDV infection. To assess this hypothesis, a GFP-expressing CRISPR/Cas9 therapeutic plasmid specifically recognizing the virus-encoded *pp38* open reading frame was constructed. This plasmid construct was then transformed into the attenuated ST strain (*Δlon ΔsifA*) for systemic delivery via infection. During the infection cycle, both the virus and ST occupy similar niches, such as macrophages, monocytes, and spleen and liver tissues [22]. Thus, ST can be used to deliver a therapeutic CRISPR/Cas9 plasmid directly into these cells and tissues, where the expressed Cas9 system may act as a molecular scissor to inactivate virus-encoded genes [23, 24]. The efficacy of plasmid delivery was investigated by assaying GFP expression in spleen and liver tissues under in vivo and in vitro cell culture conditions. The viral activity in infected animals was quantified by a qRT–PCR-based method. In addition, viral pathogenesis related to the treatment was evaluated by observing general symptoms, such as reduced body weight, reduced appetite, time to paralysis, morbidity, mortality, and cancerous transformation in visceral organs [16]. In the present study, five test groups were created: naïve control (A), PBS control (B), MDV-1^st^ (C; viral infection occurred first), ST-1^st^ (D; ST infection occurred first), and vector control (E) groups. According to our results, the ST strain showed promising potential for in vivo CRISPR/Cas9 system delivery, and treatment resulted in significant resistance against viral infection in the ST-1^st^ group, where the plasmids were delivered into target tissues via the ST strain before viral infection took place. Both ST-treated groups (C & D) showed significant resistance marked by delayed paralysis, a low reduction in body weight, an extended lifespan, and reduced visceral tumours and lesions, showing the degree of effectiveness of the CRISPR/Cas9 therapy. Even though none of the test groups showed complete recovery due to the high pathogenicity of the virus strain used in the study, the ST strain demonstrated promising potential for use as an in vivo CRISPR/Cas9 plasmid delivery platform that can be placed among other leading strategies with potential for in vivo CRISPR-mediated DNA therapy.

## Materials and methods

### Bacterial strains and plasmids

The bacterial strains and plasmids used in the present study are listed in Table 1. All *Salmonella* Typhimurium (ST) and *Escherichia coli* strains were routinely grown in Luria–Bertani (LB; Sparks, MD, USA) broth or LB agar containing the antibiotic ampicillin or kanamycin (50 μg/mL) at 37 °C with continuous agitation (195 rpm). To enhance plasmid delivery efficacy, the *sifA* gene of *Salmonella* Typhimurium was knocked out in the ST *Δlon* parental strain using a lambda red recombination procedure [25]. The flippase recognition target-flanked chloramphenicol (frt-Cat-frt) gene cassette was amplified using *sifA* flanking primers by using the pKD3 plasmid as the template. The 1.1 kb long linear fragment was then transformed into the pKD46 plasmid, and recombinase-competent ST was created by replacing the *sifA* gene. Subsequently, the chloramphenicol gene cassette was evicted by transformation of the pCP20 plasmid that encodes the flippase enzyme. The complete *sifA* gene deletion was confirmed using internal *sifA* primers.

**Table 1:** **Bacterial strains, plasmids, and primers used in this study**.

Strain/plasmid/primer	Description
Bacteria	
JOL909	*S. *Typhimurium *Δlon* (Lab stock)
JOL2030	*S. *Typhimurium *Δlon ΔsifA*
*E. coli* DH5α	F-Φ80lacZM15 (lacZYA-argF) U169 recA1 endA1 hsdR17(rK)^−^, mK^+^ phoA supE44 thi-1 gyrA96 relA1 λ ^–^ (Promega)
Plasmids	
pKD3	Template for *frt-Cat-frt* gene cassette
pKD46	Encodes recombinase enzyme
pCP20	Encodes flippase enzyme
LentiCRISPR V2	Provides CRISPR/Cas9 gene cassette
CRISPR::*pp38*	CRISPR/Cas9 system targeting *pp38* gene
CRISPR::*meq*	CRISPR/Cas9 system targeting *meq* gene
Primers	
FP *sifA* (*sifA* deletion)	TTGGTAAGGATTTCACTTTTTAAAAAACCATTCCCTATAGTAATCGGCAT GTGTAGGCTGGAGCTGCTTC
RP *sifA* (*sifA* deletion)	AGTACGTGAGTAAACCCTGAACGTGACGTCTGAGAAAGCGTCGTCTGATT ATGGGAATTAGCCATGGTCC

### Construction of CRISPR/Cas9 plasmid vectors

For plasmid tracking, purpose CRISPR/Cas9 components were fused with the backbone of the pACGFP plasmid. The CRISPR/Cas9 components were retrieved from the lentiCRISPR v2 plasmid by digestion with BamH1 and KpnI restriction enzymes and cloned into the pACGFP plasmid using the same restriction sites. To construct sgRNAs targeting *pp38* of MDV, *pp38* [(NC_002229.3:c127787-126421 complete genome Gallid herpesvirus 2 (Additional file 1)]. The sequence was subjected to in silico screening using the CRISPOR tool [26]. The sequence 5’-GAGCTTGCCCAGCAGTGCGA AGG-3’ was used as the sgRNA and synthesized (Bioneer, Daejeon, Korea). The predicted sgRNA was assessed for specificity and predicted “indel” incorporation with frameshift mutations. The predicted sequence was blasted (National Center for Biotechnology Information, Basic Local Alignment Search Tool; NCBI BLAST) against the *Gallus gallus* genome for homology prediction. The 20 nucleotide sequences were synthesized and cloned into the filler region of the CRISPR/Cas9 gene cassette utilizing the ESP3I restriction site. The insertion of the sgRNA into the filler region was confirmed by polymerase chain reaction (PCR) (data not shown).

### In vitro and in vivo plasmid delivery

To investigate in vitro plasmid delivery efficacy, Caco2 cells were either infected by the ST strain harbouring CRISPR/Cas9 at 100 or 150 MOI or transfected using lipofectamine [LyoVec (InvivoGen, San Diego, CA, USA)] with a variable plasmid concentration of 5 µg, 10 µg, or 15 µg per well. The morphology and expression of green fluorescent protein (GFP) were observed under an IncuCyte imaging system (Essen BioScience, Ann Arbor, MI, USA). The total number of green fluorescent objects and the confluence level were quantified using the IncuCyte metric graph option. In vivo plasmid delivery efficacy was evaluated in a group of chickens (5 weeks old). Three groups (*n* = 4) of birds were infected with ST strains harbouring the CRISPR/Cas9::*meq* plasmid at a dose of 2 × 10^8^ CFU/100 μL/bird via the intraperitoneal route. Two groups (*n* = 3) were maintained as the PBS control and naïve control groups. On the 3^rd^, 6^th^, and 7^th^ days post-inoculation, birds were euthanized, and spleens were aseptically harvested for splenocyte preparation. Harvested cells were analysed for green fluorescence in a fluorescence-activated cell sorting (FACS) assay. The PBS and naïve groups were treated as negative controls.

### Cell lines, animals, and experimental infection

**Figure 1:**
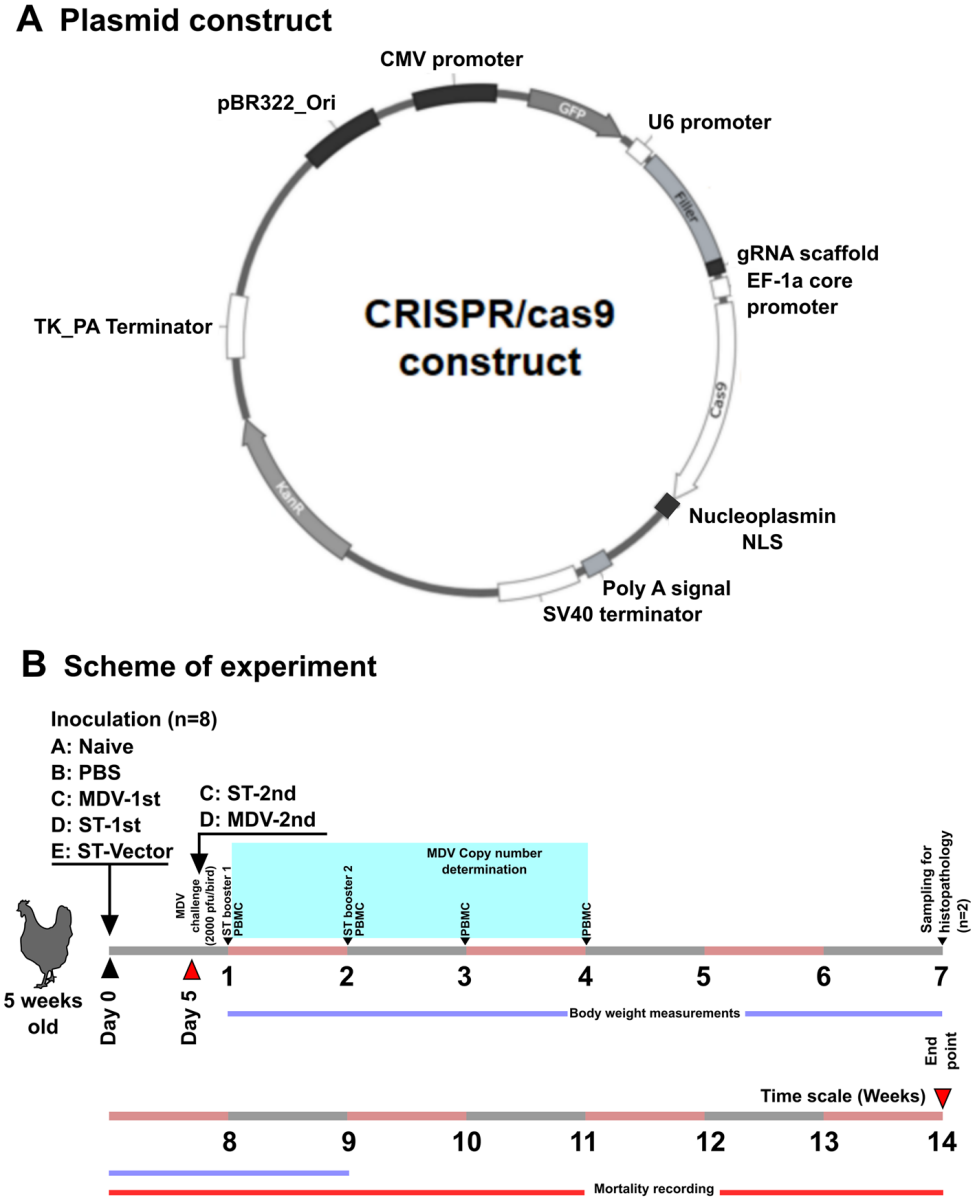
**Plasmid design and general experimental scheme.A** Schematic representation of the CRISPR/Cas9 plasmid construct. Major elements including (1) Tk_PA_terminator, (2) pBR322_origin, (3) CMV promoter, (4) GFP, (5) U6 promoter, (6) filler, (7) gRNA scaffold, (8) Ef-1a core promoter, (9) Cas9, (10) nucleoplasmin NLS, (11) poly A signal, and (12) SV40 terminator have been depicted. Note: elements are not depicted in proportion to their size. **B** Schematic representation of the experimental scheme. On Day 0, one-month-old birds were treated as follows: A, naïve; B, PBS; C, MDV-1^st^; D, ST-1^st^; and E, ST vector. On Day 5, the birds in Groups C and D were treated with ST and MDV, respectively (treatments were switched). On Days 7 and 14, ST boosting was conducted. At 6^th^ week post-challenge, two birds from each group were euthanized and used for histopathological analysis by H & E staining. Body weight measurements were conducted until the 9^th^ week, and mortality was observed until the 14^th^ week post-immunization.

A strain of the oncogenic Marek’s disease virus (MDV), also known as Gallid herpesvirus 2, was purchased from American Type Culture Collection (Strain 648A; ATCC VR-1576). Virus propagation was conducted with a chicken embryonic fibroblast cell line purchased from ATCC (CRL-1590) routinely grown in Dulbecco’s modified Eagle’s medium (DMEM; Bio Whittaker, Walkersville, MD, USA) supplemented with 10% foetal bovine serum (Serana, Dorfstrasse, Pessin, Germany) and 5% tryptase phosphate broth (Sigma–Aldrich, St. Louis, MO, USA) in a humidified 5% *CO*_*2*_ atmosphere at 37 °C. Briefly, the chicken embryonic fibroblast cell line was propagated in the prescribed medium in 75 cm^2^ cell culture flasks until reaching approximately 80–90% confluence. Then, a virus-containing vial was thawed in a 37 °C water bath with gentle agitation. After thawing, the vial was decontaminated with 70% ethanol, and the total content was added to 9 mL of complete medium and spun at 125×*g* for 10 min. The supernatant was decanted, and the pellet was resuspended in 10 mL of complete medium and overlaid in a 75 cm^2^ cell culture flask. Cells were incubated at 37 °C until visible cytopathy was observed before subculture. The animal experiment was approved by the Chonbuk National University Animal Ethics Committee (accession number: CBNU2018-00264), and the chicken experiment was carried out according to the guidelines of the Korean Council on Animal Care. One-day-old female Brown Nick layer chickens (Corporation of Join hatchery, Republic of Korea) were maintained under standard conditions and provided food and water ad libitum. At five weeks of age, chickens were randomly divided into five groups (*n* = 8) named Group A, naïve; Group B, PBS; Group C, 1^st^ –MDV infected; Group D, 1^st^—*Salmonella* (ST) infected; and Group E, vector control. To infect chickens with MDV, the fifth passage of chicken embryonic fibroblasts (CEFs) was subcutaneously injected into test birds (Group C; 2000 pfu/bird/200 µL PBS). Birds from Groups D and E were infected with 2 × 10^8^ CFU/bird inoculum containing ST harbouring CRISPR::*pp38* or the naked vector, respectively (Figure 1B). After five days, all birds except those in Groups A and C were infected with 2000 pfu of MDV. The titration of MDV was performed by considering the TCID_50_ (pfu = TCID_50_ × 0.7) value determined in 96-well plates following a standard TCID_50_ determination procedure [27]. Briefly, CEFs were seeded in 96-well plates in complete medium and allowed to reach 90% confluence. Cells infected with MDV were trypsinized and collected by centrifugation. The cells were diluted tenfold and overlaid in 96-well plates (8 wells/dilution). The plates were observed daily for signs of cytopathy, and after ten days, the plates were used to determine the TCID_50_ value [28]. Furthermore, copy numbers were verified by a qRT–PCR method involving determination of the CT value for the *meq* gene. The copy number was derived by a previously established relationship [29]. pfu numbers were derived by multiplying the TCID_50_ value by a factor of 0.7. Birds were closely monitored for symptom development. Body weight measurements were performed once per week. Booster immunization with ST was conducted on the 7^th^ and 14^th^ days post-primary MDV infection for the birds in Groups C and D with a dose similar to that use for the primary inoculation.

### In vitro detection of mutations

To check the preservation of the CRISPR activity of the plasmid construct, an in vitro transfection study was conducted. MDV-infected chicken fibroblasts (5^th^ passage) were seeded in 96-well plates and grown until 70% confluent in complete medium. Varying concentrations of the CRISPR::*pp38* plasmid (0.2, 0.4, 0.6, 0.8 and 1 μg/well) were used following the manufacturer’s procedure. The cells were further incubated for 24 h, and total genomic DNA was isolated. The whole *pp38* gene segment was amplified by PCR and subjected to gel purification. The initially obtained enzyme-resistant band was gel purified and reamplified. Next, extracted DNA was digested with the restriction endonuclease PspFI, and DNA with loss of the restriction site was detected on a 0.8% agarose gel. Uncut DNA was then cloned into a TA vector and sequenced. The deletion 4 nucleotide segment could be identified.

### Sample collection and RNA extraction

Blood samples were collected from experimental birds via the wing veins following a standard procedure on the 7^th^, 14^th^, 21^st^, and 28^th^ days post MDV infection. Approximately 4 mL of blood was collected from each bird and directly dispensed into 15 mL conical tubes containing 2 mg/mL ethylenediaminetetraacetic acid (EDTA; Sigma, St. Louis, Missouri, USA). Following blood collection into EDTA-treated collection vessels, peripheral blood mononuclear cells (PBMCs) were harvested within approximately 2 h of collection using Histopaque-1077 (Sigma–Aldrich, St. Louis, MD, USA). Briefly, 1 mL of Histopaque-1077 was added into 2 mL centrifuge tubes, and then an equal volume of blood was carefully dispensed onto the Histopaque-1077 layer. After centrifugation at 1600 RPM and room temperature for 30 min, the white translucent band was collected. The cell number was determined using a haemocytometer, and 1 × 10^6^ cells were used to extract RNA using a commercial RNA extraction kit (GeneALL; Songpa-gu, Seoul, Korea) according to the recommended procedure. RNA purity was assessed by determining A260/A230 absorbance measurements using a microplate reader (Tecan, Mannedorf, Switzerland), and all ratios were approximately 2.0. Extracted RNA was immediately reverse transcribed into cDNA using a ReverTra Ace kit (Toyobo, Osaka, Japan) and stored at −20 °C until further use in a qRT–PCR assay.

### Quantitative real-time PCR

Quantitative real-time PCR (qRT–PCR) was performed using SYBR green to determine the viral copy number. Herein, the *meq* gene of MDV was selected as the reference gene, and the *ovo* gene was used as the housekeeping gene [30]. The primer sequences for the *meq* gene (P1: 5’-CCCAACAGCCCCTCCAAACAC-3’ and P2: 5’-CTTCATGGAGTTTGTCTACA-3’) and the *ovo* gene (P3: 5’-CACTGCCACTGGGCTCTGT-3’ and P4: 5’-CACACACAAAAACCCAGCCT-3’) were retrieved from a previous description [29] and chemically synthesized (Bioneer, Daejeon, South Korea). qRT–PCR was performed on a StepOnePlus real-time PCR system (Applied Biosystems; Waltham, MA, USA). The Ct values obtained by qRT–PCR were then converted into viral copy numbers present in PBMCs using a previously established relationship [29].

### Assessment of body weight, morbidity, and mortality

To assess MDV-inflicted pathogenesis in test animals, body weight measurements were taken weekly for 10 weeks post-viral infection. Morbidity and mortality incidences were also recorded.

### Histopathological examination

Histopathological assessment of spleen, liver, lung, and intestine specimens was conducted by haematoxylin and eosin (H & E) staining and microscopic examination as previously described [31]. Briefly, aseptically harvested spleen and liver specimens were fixed in a 10% formaldehyde solution for 3 days at 4 °C. Then, the tissue specimens were cut into appropriately sized samples and dehydrated with a graded ethanol series. Next, the samples were embedded in paraffin, and 10-µm-thick sections were cut using a microtome and stained according to the standard procedure.

### Assessment of serum cytopathy

The infectivity of MDV-containing serum was tested by monitoring cytopathy in cultured chicken fibroblasts. Chicken serum collected on the 5^th^ week of post-challenge was assessed. Microscopic observation of cellular cytopathy was compared among the groups. Collected serum samples were diluted 1:50 in serum-free medium and incubated for 1 h and 30 min. Cells were washed with PBS three times and replaced with complete medium. After 6 days of incubation, the cells were observed to assess the induced cytopathic effect.

### In vivo detection of mutated DNA

To confirm that the MDV genome was mutated by the ST-delivered CRISPR plasmid under in vivo conditions, RNA isolated from PBMCs was tested for resistance to the restriction enzyme PspFI. Total RNA was isolated from PBMCs collected from the ST-1^st^ group and used for analysis. A pooled cDNA sample was prepared using cDNA prepared from PBMCs collected weekly from six birds. The cDNA sample was serially diluted, and PCR amplification of *pp38* was performed. The gene band was excised, purified, and subjected to RE digestion with the enzyme PspF1. Positive PCR samples were reamplified and used for secondary confirmation. Selected specimens were digested with PspF1 and resolved in an agarose gel. The proportion of resistant gene products with an approximate size of 1.3 kb was compared to that of the digested gene products with approximated sizes of 0.9 kb and 0.4 kb.

### Statistical analysis

All data were analysed using GraphPad Prism 6.00 (San Diego, CA, USA). One-way analysis of variance (ANOVA) with Tukey’s multiple comparison test was conducted to determine the significance of differences among vaccinated and control groups. A difference was considered significant at a *P* value < 0.05.

## Results

### Propagation of Marek’s disease virus

The initial propagation of MDV (ATCC; VR-1576) was carried out with cultured chicken fibroblasts (ATCC; CRL-1590). To expedite MDV propagation in subsequent passages, chicken kidney cells were also used. Determination of the viral titre was performed by the TCID_50_ method relying on the MDV-induced cytopathic effect [32–34], and the viral titre (in pfu/mL) was determined by multiplying the TCID_50_ value by 0.7 or from the absolute copy number determined from the qRT–PCR-derived CT value for the *meq* gene. The titres derived by the two methods were comparable and calculated to be 1.624 × 10^7^ pfu/mL and 1.879 × 10^7^ pfu/mL, respectively. For chicken infection, virus-infected chicken fibroblasts were collected at the 7^th^ passage, and approximately 2000 pfu/chicken/200 µL of culture medium was used as the subcutaneous inoculation dose.

### Plasmid construction and detection of mutations in vitro

**Figure 2:**
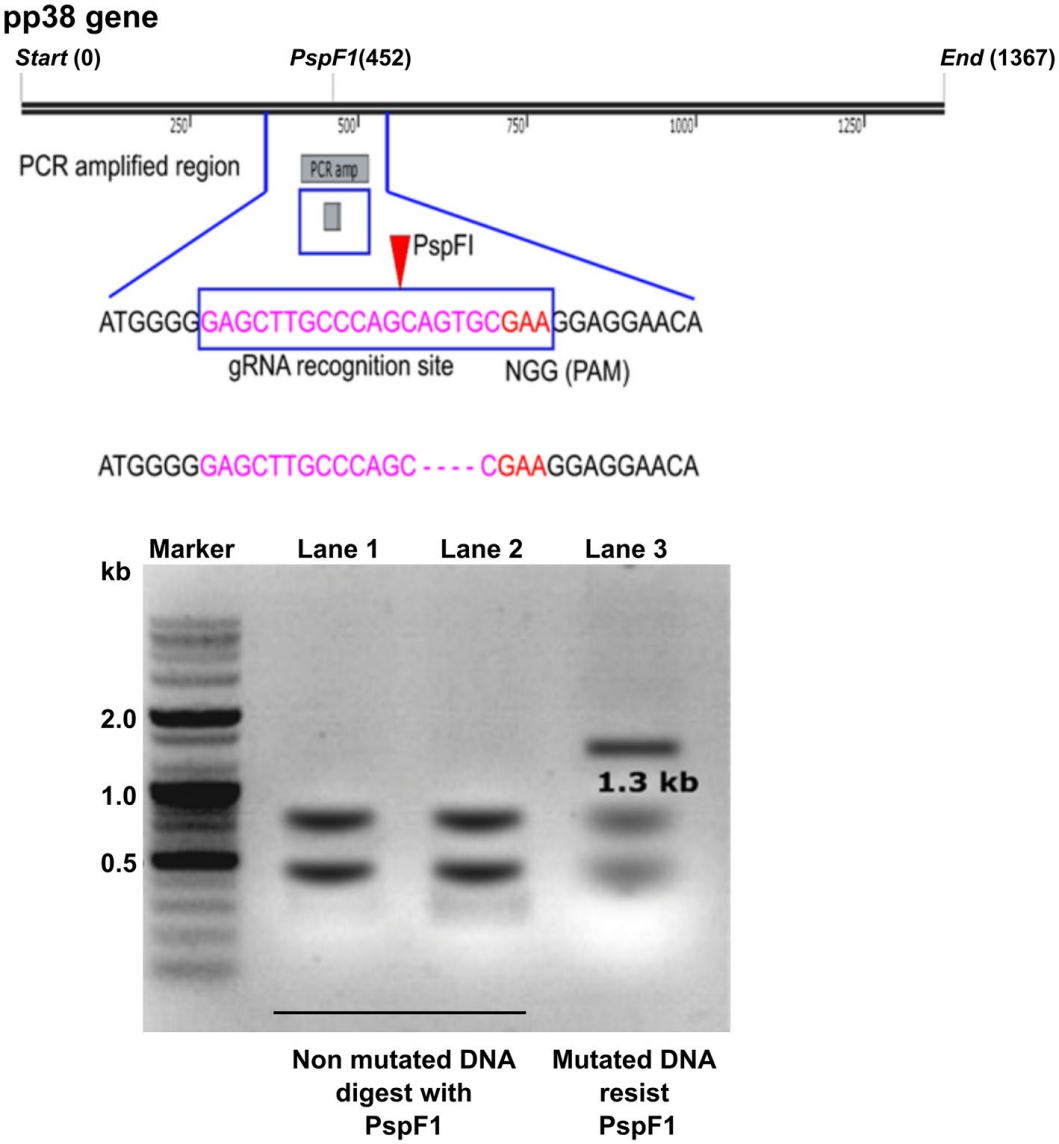
**In vitro detection of CRISPR-mediated mutations**. To confirm the activity of CRISPR-mediated targeted gene mutation, chicken fibroblasts were seeded in 96-well plates. Varying concentrations of CRISPR::*pp38* plasmid DNA (0.2, 0.4, 0.6, 0.8 and 1 μg/well) were transfected using a lipofection procedure. The cells were incubated in complete DMEM for 36 h. Genomic DNA was isolated, and the target *pp38* gene was amplified by PCR. The gel-purified DNA fragment was digested with the restriction enzyme PspF1. Uncut DNA was extracted, cloned into the TA vector, and sequenced to detect the frameshift mutation.

The *pp38*-specific sgRNA was scored for specificity and frameshift-indel mutations using the CRISPOR tool. The MIT specificity score was 100, while the out-of-frame score and Lindel score were 79 and 86, respectively. Zero off-targets were predicted. The BLAST query against the *Gallus gallus* genome resulted in a maximum of 76% sequence identity (query cover Χ percent identity) and was considered reasonable to undertake the experiment. The CRISPR plasmid construct CRISPR::*pp38* (Figure 1A) was transformed into the attenuated ST (*ΔlonΔsifA*) strain and used to infect experimental chickens. The preserved Cas9 activity and indel mutation formation ability of the plasmid were further confirmed in an in vitro assay using MDV-infected chicken fibroblasts treated with the CRISPR::*pp38* plasmid. After transfection of the plasmid construct, a frameshift deletion could be detected by monitoring the loss of the PspFI restriction site in the vicinity of the sgRNA-PAM construct (Figure 2). The gene segment was amplified by PCR and digested with the restriction enzyme PspFI, and then the uncut DNA was gel purified and cloned into a TA vector for sequencing. Having found that the plasmid is capable of causing indel mutations [35], a similar activity can also be expected in vivo in virus-infected chickens.

### In vitro and in vivo plasmid delivery efficacies

**Figure 3:**
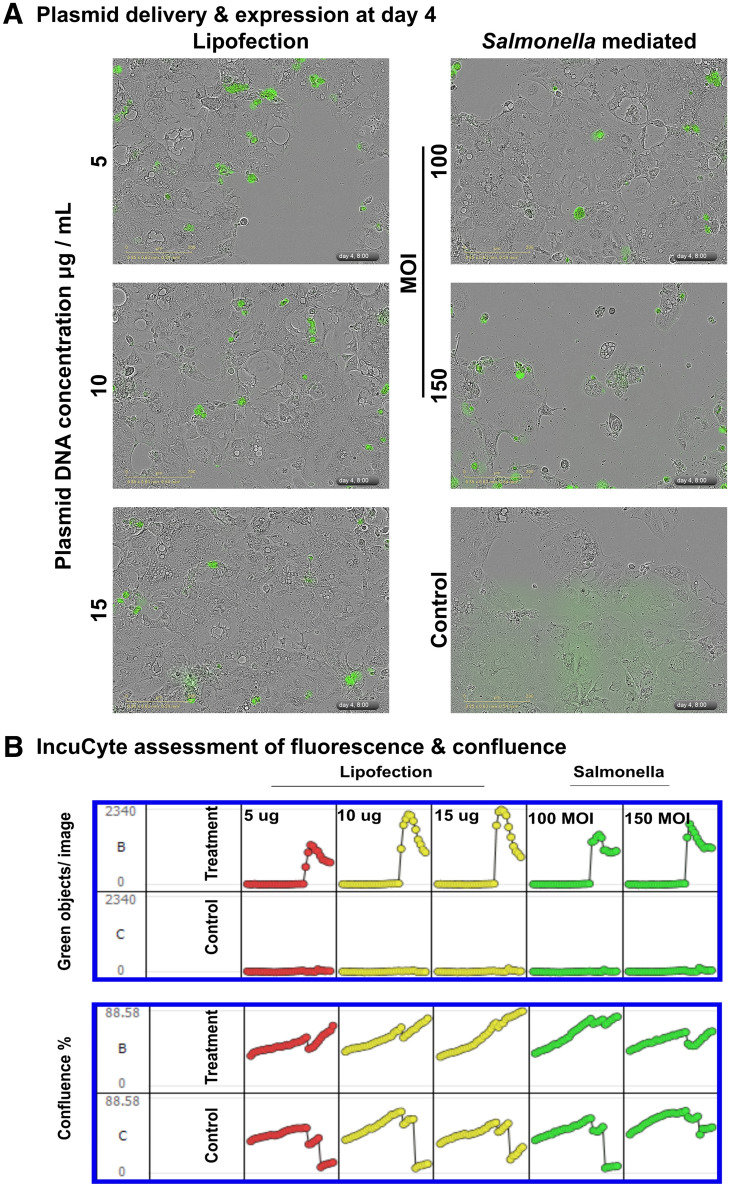
**In vitro plasmid delivery efficacy.A** Caco2 cells were transfected with the CRISPR/Cas9 plasmid via either lipofection with variable plasmid concentrations (5, 10, or 15 μg/well) or ST-mediated bacterial transfection with variable MOIs (100 and 150) and observed using an IncuCyte live imaging system for six days. Images acquired on Day 4 are shown. The development of green fluorescent objects was observed and quantified. **B** The number of green objects per image per well and the confluence levels measured over six days are depicted. Two independent trials were performed, and representative images are shown. The arrow indicates the time of plasmid introduction or ST infection.

**Figure 4:**
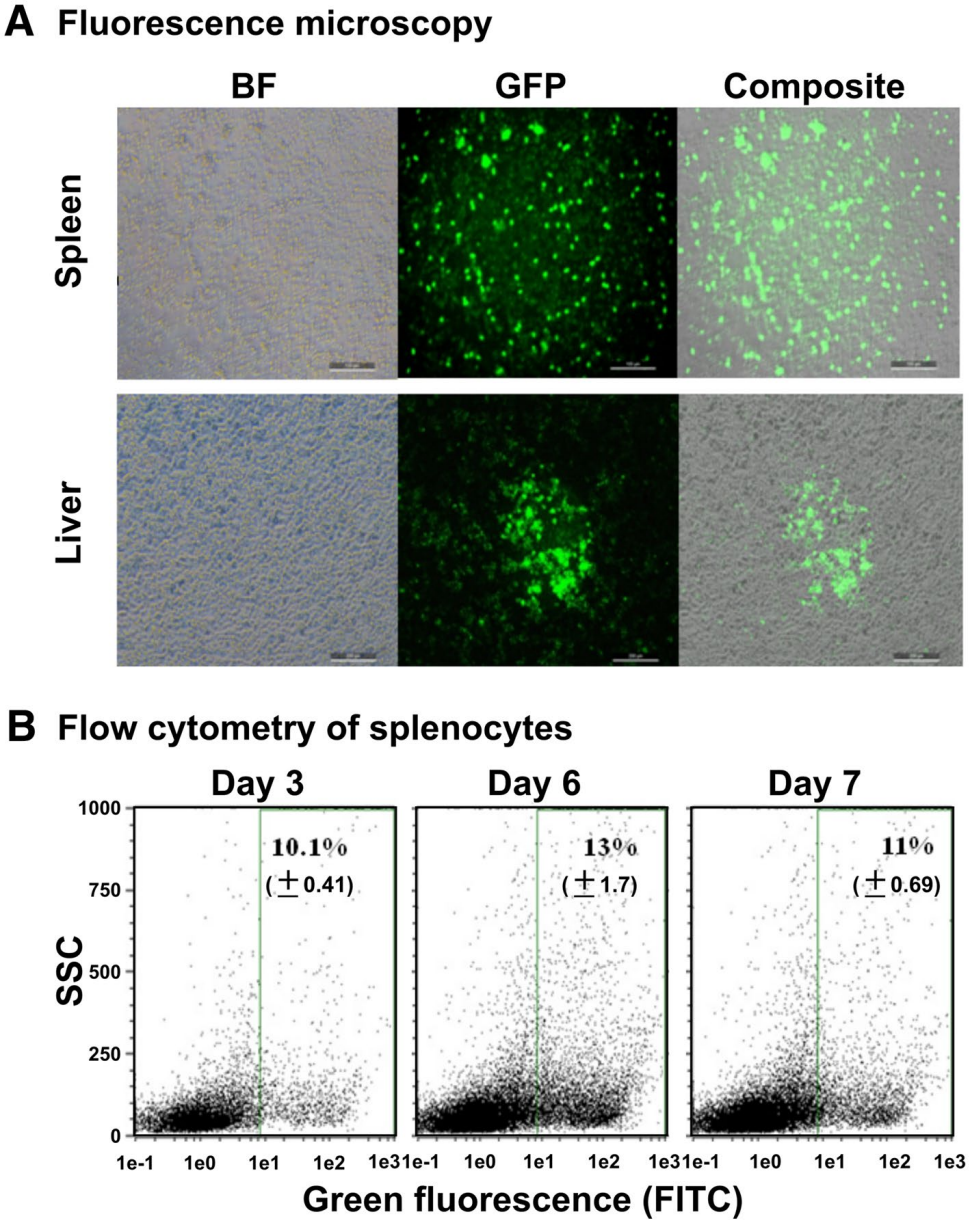
**In vivo plasmid delivery efficacy**. **A** Fluorescence microscopy examination of splenocytes and liver cells harvested on the 6^th^ day post ST treatment. BF, bright field; GFP, green fluorescent protein; composite, merged composite image. **B** FACS-based quantification of plasmid delivery in splenocytes harvested on the 3^rd^, 6^th^, or 7^th^ day post ST treatment. The maximum expression of plasmids was observed on the 6^th^ day post-treatment. An average of three trials was performed, and representative scatter plots are shown.

*Salmonella-*mediated in vitro plasmid delivery efficacy was compared with the efficacy of a conventional lipofection procedure using an IncuCyte live imaging system. Green fluorescence signals could be detected on the 4^th^ day onward for both infected and ST-transformed cells. The total number of green fluorescent objects per image showed an incremental pattern depending on the plasmid DNA concentration and the multiplicity of infection of the ST strain (Figures 3A and B). To assess the in vivo plasmid delivery efficacy, spleen and liver specimens were collected, and single cells were harvested for a FACS-based assay to quantify the extent of green fluorescence-positive cells at different time points (3^rd^, 6^th^, and 7^th^ days post-inoculation). Within 3 days, ST populated the spleen and liver; thus, random delivery and expression of GFP occurred. The highest GFP-expressing cell population (13 ± 1.7%) was found in splenocytes collected on the 6^th^ day post-inoculation, but this population subsequently diminished to 11 ± 0.69% on the 7^th^ day (Figures 4A and B). Plasmid delivery efficacy in liver cells varied, reaching 8.0 ± 1.8% on the 6^th^ day. The expression of GFP in splenocytes and liver cells confirmed the successful release of the plasmid cargo by therapeutic *Salmonella*. The plasmid was segregationally stable over five days of investigation without significant loss during growth without antibiotic selection pressure.

### Body weight, morbidity, and mortality of animals

**Figure 5:**
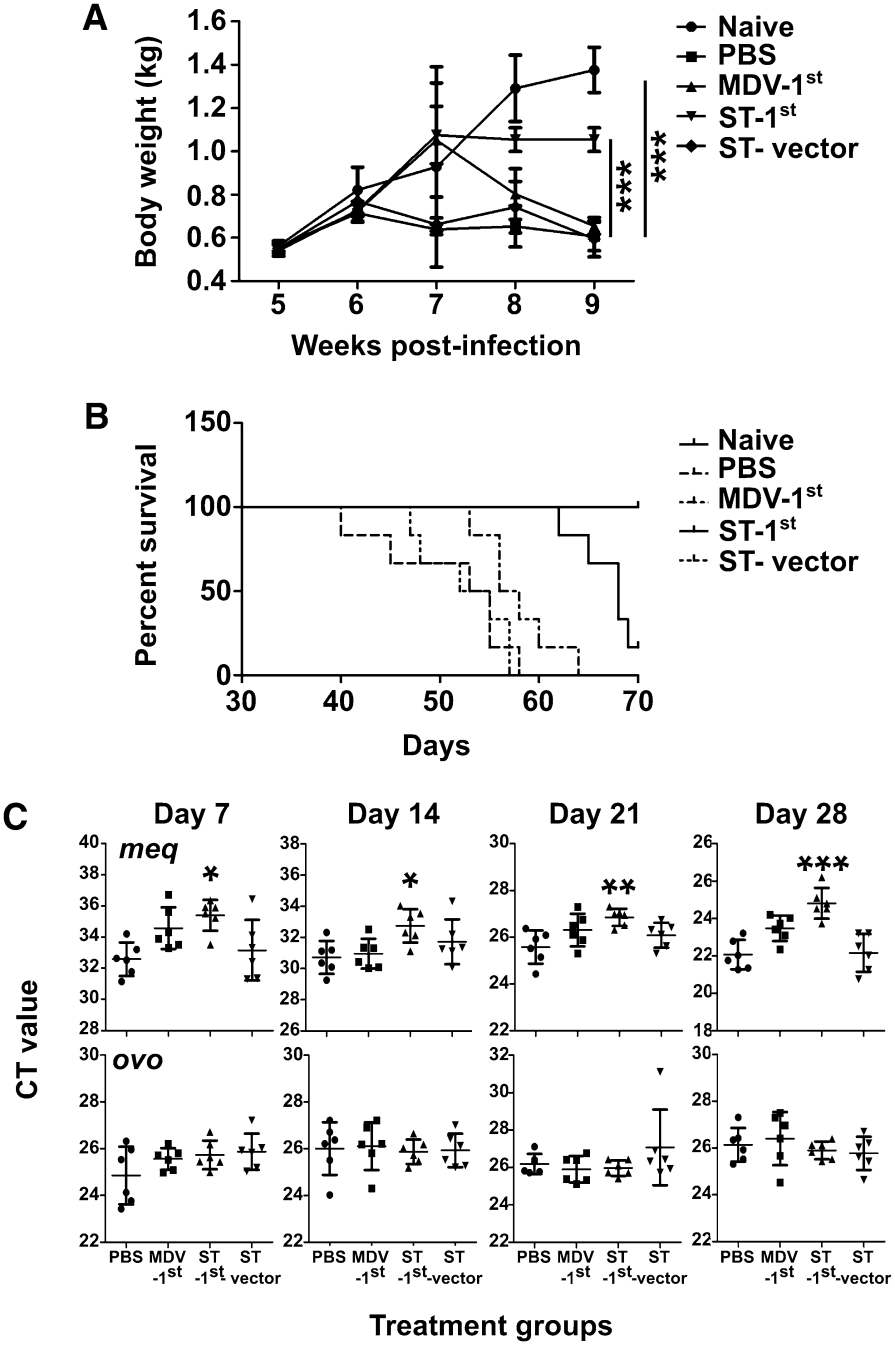
**Body weight, survival, and viral copy number of chickens after MDV challenge**. **A** The body weight of chickens (*n* = 6) in all five groups was measured once a week for 9 weeks. Compared to the other groups, the ST-1^st^ group demonstrated significant resistance against MDV infection and showed the smallest weight loss. *** indicates *p* < 0.05. **B** The survival rate of chickens in each test group. The chickens belonging to the ST-1^st^ treatment group exhibited extended survival compared with those in the other groups upon MDV infection. **C** The qRT–PCR-measured changes in the threshold cycle (Ct) values of the *meq* and *ovo* genes in the chicken groups undergoing experimental MDV infection. Total RNA was extracted from PBMCs collected on the 7^th^, 14^th^, 21^st^, or 28^th^ day post MDV infection for each chicken group, and cDNA was synthesized. The circulating MDV viral load was determined by a qRT–PCR-based assay using the *meq* gene as the reference gene. An increasing MDV viral load was demonstrated by reduced Ct values in all samples with time. The viral loads in the ST-1^st^ group showed significant differences beyond Day 21 post-infection. The change in the Ct values of the internal housekeeping *ovo* gene is shown. *** indicates a significant difference compared to the PBS control group. *P* < 0.05. The average CT values and standard deviation of three independent trials are shown.

Body weight, morbidity, and mortality were monitored from the 5^th^ week to the 9^th^ week after viral infection in the animal groups. At 5 to 7 days post-infection with the virus, all chickens demonstrated temporary signs of paralysis, with return to a normal state between 24 and 48 h after the occurrence. Intraperitoneal injection of attenuated ST did not produce a significant disease condition, such as reduced feed intake, diarrhoea, or lethargy. However, beyond the 7^th^ week, a drastic reduction in body weight was observed in Group B, C, and E animals (Figure 5A), possibly due to viral activity and pathogenesis. At the end of the 9^th^ week, the body weight of the remaining animals in Group B was 33% of that of the noninfected animals. Complete paralysis and death of some animals were also evident after the 8^th^ week in Groups B, C, and E (Figure 5B). Comparatively, Group D animals demonstrated significant resistance with minimal weight reduction and an extended time to complete paralysis. Compared to the chickens in the control groups, all six individuals in Group D lived for more than 90 days longer after primary Marek’s disease virus infection (Figure 5B).

### Quantification of the viral genome in PBMCs from infected animals

To investigate changes in viral activity in test animals, a SYBR green qRT–PCR-based method was adapted. The presence of the viral genome in the peripheral blood was assessed by harvesting peripheral blood mononuclear cells (PBMCs) from each bird at a two-week interval. Total RNA was extracted from 10^5^ PBMCs collected from each animal, and cDNA was synthesized. The viral load was assayed using the *meq* gene as the marker gene for the viral genome and the chicken *ovo* gene as the internal control. The threshold cycle (CT) value obtained from each reaction was then converted into a viral copy number using the previously described relationship Y (Ct value) = 39.732–3.089 X (log concentration) for the *meq* gene and Y = 40.172–3.053 X for the ovo gene. The copy number was calculated per 10^5^ PBMCs. The results demonstrated a significant reduction in viral copy numbers in Group D animals, which were given ST treatment before virus infection, by the 28^th^ day post-infection (Figure 5C). Furthermore, virulence assessment of MDV-containing serum showed observable virulence reduction in Group D chickens (Additional file 2), suggesting the possible occurrence of attenuation in CRISPR-treated chickens. These observations corroborated the viral copy number assessment results, suggesting the importance of timing in the delivery of the ST-CRISPR/Cas9 system before MDV infection (Table 2).

**Table 2:** **Quantification of the MDV genome in peripheral blood mononuclear cells from chickens in the test groups (mean ± SD,*n*** **= 6)**.

Day	Gene	Group				
		A	B	C	D	E
7	*meq*	Nd	2.7 ± 1.9	0.6 ± 0.3	0.3 ± 0.3	2.5 ± 2.3
	*ovo*		1446.1 ± 1140.8	644.2 ± 222.1	583.6 ± 240.5	549.1 ± 270.0
14	*meq*	Nd	10.6 ± 7.9	8.4 ± 4.4	2.4 ± 2.0	5.6 ± 4.1
	*ovo*		620.5 ± 670.7	537.5 ± 518.9	512.7 ± 197.4	517.1 ± 244.1
21	*meq*	Nd	431.9 ± 254.6	247.5 ± 127.4	490.4 ± 13.9	591.3 ± 343.8
	*ovo*		442.6 ± 212.9	456.5 ± 459.3	490.4 ± 13.9	591.3 ± 343.8
28	*meq*	Nd	5941.1 ± 2975.2	2060.1 ± 1174.5	*777.4* ± 430.0^*^	6156.8 ± 4637.6
	*ovo*		404.1 ± 140.2	525.7 ± 259.1	468.1 ± 259.1	315.2 ± 186.7

Gene copy number × 10^3^/10^5^ cells.

The chicken was subjected to *Salmonella*-mediated CRISPR treatment and experimental infection with MDV as follows. A: Naïve, B: PBS control, C: 1^st^—MDV infection, D: 1^st^—*Salmonella* infection, E: vector control. Viral copy numbers were determined in peripheral blood mononuclear cells (PBMCs) on the 7^th^, 14^th^, 21^st^, and 28^th^ days post-treatment. Using the meq gene (target) and the ovo gene (housekeeping gene) viral copy numbers were determined and each group was compared against the PBS control for each collection day. A significant reduction in viral copy numbers was observed only in group D birds by the 28^th^ day compared to the PBS control group. This result highlights the importance of timing of *Salmonella* treatment to significantly reduce the impact of MDV infection. Before the MDV infection, *Salmonella* infection must take place to deliver the CRIPSR plasmid for effective interception of viral genome. The level of significance was determined if the *p* < 0.05 (in bold italic).

^*^Indicates a significant difference in viral copy number compared to the PBS control group (*p* < 0.05).

### Observations in visceral organs

**Figure 6:**
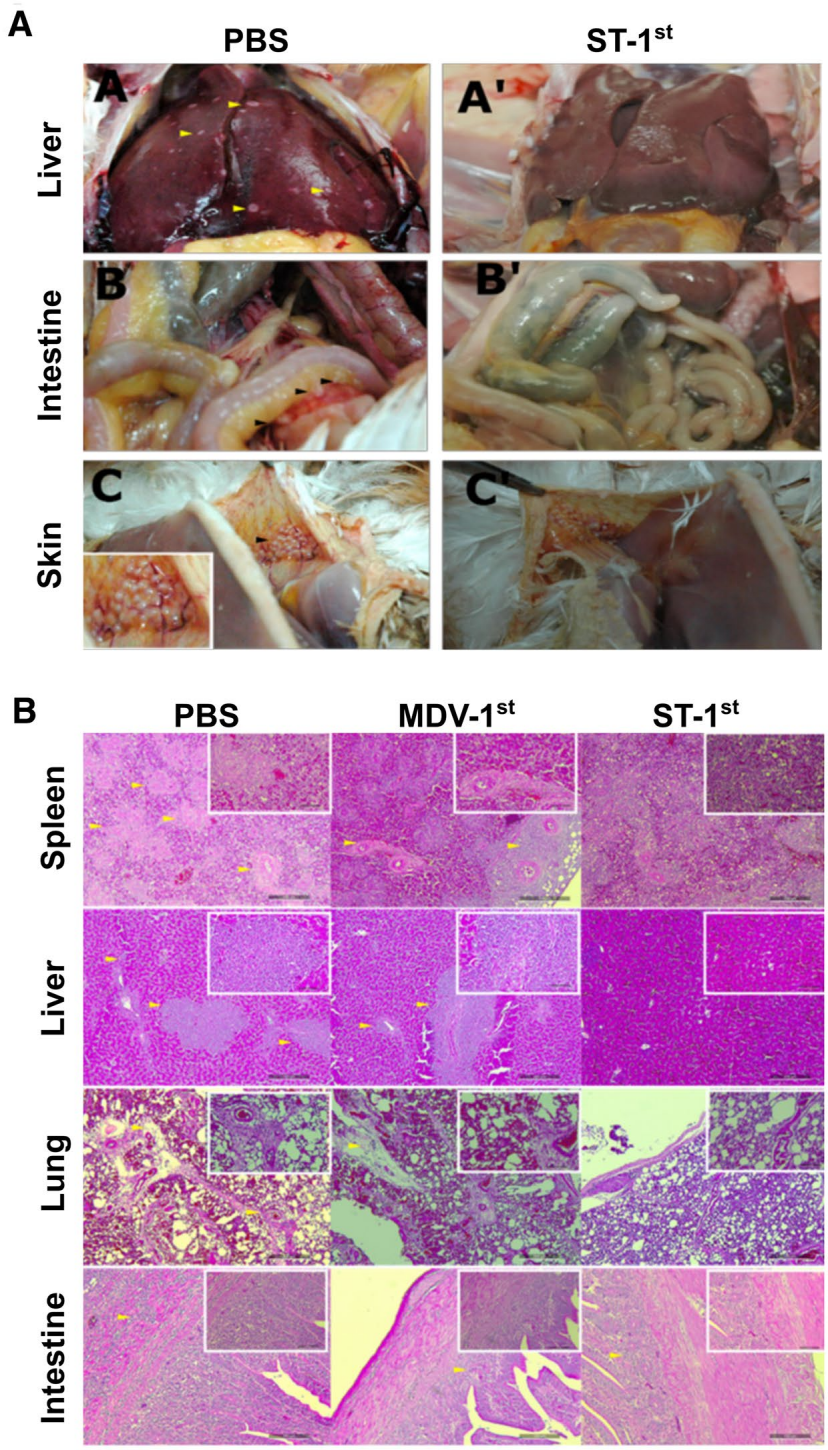
**Marek’s disease virus-induced pathological consequences in chickens.A** A comparison between the PBS control and ST-1^st^ treatment groups. Livers from the PBS control (A^A^) group showed MDV-induced lesions, whereas those from the ST-1^st^ group did not show severe lesions (A^A’^). In the intestine of the PBS control group animals, cancerous tumours were visible (A^B^), but tumours were absent in the ST-1^st^ group (A^B’^). Tumour-like projections were visibly attached to feather bases in both groups (A^C^ & A^C’^). **B** Histopathological examination. Organ specimens were collected from infected chickens in each group (*n* = 2) at 6 weeks post-infection and subjected to H & E staining. MDV-induced cancerous transformation was notably evident in the spleen and liver. Aggregation of infiltrated lymphocytes was prominent in lung and intestinal tissues. Delayed symptom development was evident in the ST-1^st^ group, showing the effectiveness of CRISPR/Cas9 delivery before MDV infection. Yellow arrowheads depict MDV-induced cancerous transformation (spleen and liver) and lymphocyte aggregation in the lungs and intestine.

Virus-induced oncogenesis was investigated in experimental animals. At six weeks post-viral infection, 2 birds from each group were sacrificed, and the visceral organs were observed (Figure 6A). Prominent virus-induced lesions were observed in the spleen of control group animals (Groups B and E), which showed severe damage (Additional file 3), whereas groups treated with the ST-mediated CRISPR/Cas9 system showed reduced tissue damage.

### Histopathological examination

Virus-induced lesions in spleen and liver specimens could be identified by the prominent patchy appearance in tissue sections (Figure 6B). Signs of inflammation and immune cell infiltration could also be observed but were less prominent in spleen and liver tissues than in lung specimens. A clear reduction in virus-induced tissue damage was observed in Group D (ST-1^st^), especially in liver tissues from infected animals.

### In vivo detection of pp38 gene mutation

**Figure 7:**
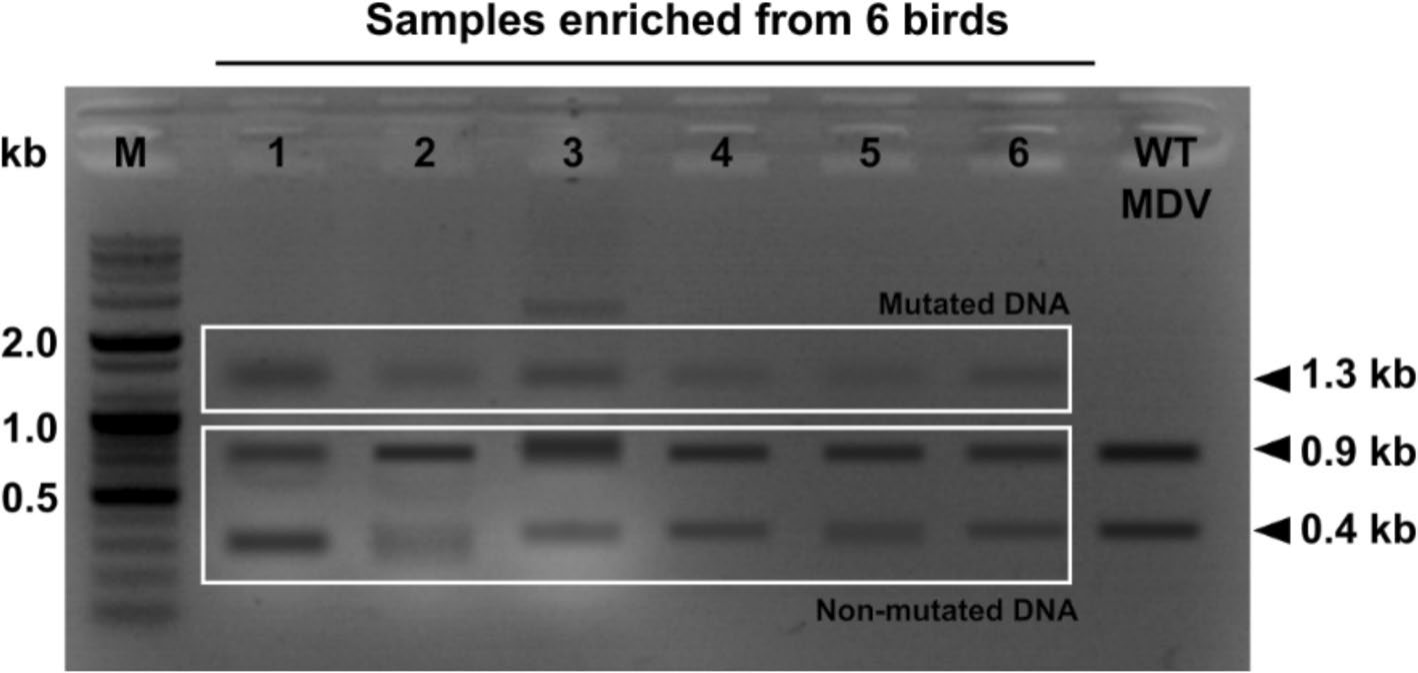
**In vivo detection of *pp38* mutation.** Loss of the PspF1 restriction site was detected in cDNA constructed using RNA templates from PBMCs. PBMCs were collected from the ST-1^st^ group, and total RNA was isolated and used to synthesize cDNA. The *pp38* ORF was amplified by PCR using cDNA as the template. Positive samples were excised from gels, reamplified and digested with the restriction enzyme PspF1. Resistant DNA demarcated the loss of the restriction site due to ST-mediated CRISPR interference. The *pp38* ORF from wild-type MDV was treated as the control.

In vivo detection of mutations was performed by evaluating the loss of the PspF1 restriction site located in the CRISPR sgRNA-targeted region of the *pp38* gene using RNA isolated from PBMCs. Due to systemic infection, MDV could be detected in PBMCs; therefore, PBMC samples were collected from the ST-1^st^ group (group with the best response induced by ST-CRISPR treatment). The cDNA prepared from weekly samples used for RNA isolation was pooled together and used for *pp38* ORF amplification in serial dilutions. Positive specimens were excised from gels and reamplified for restriction digestion with the enzyme PspF1. An enzyme-resistant DNA fragment with a size of 1.3 kb resembled a mutated gene product created by ST-mediated CRISPR treatment (Figure 7).

## Discussion

**Figure 8:**
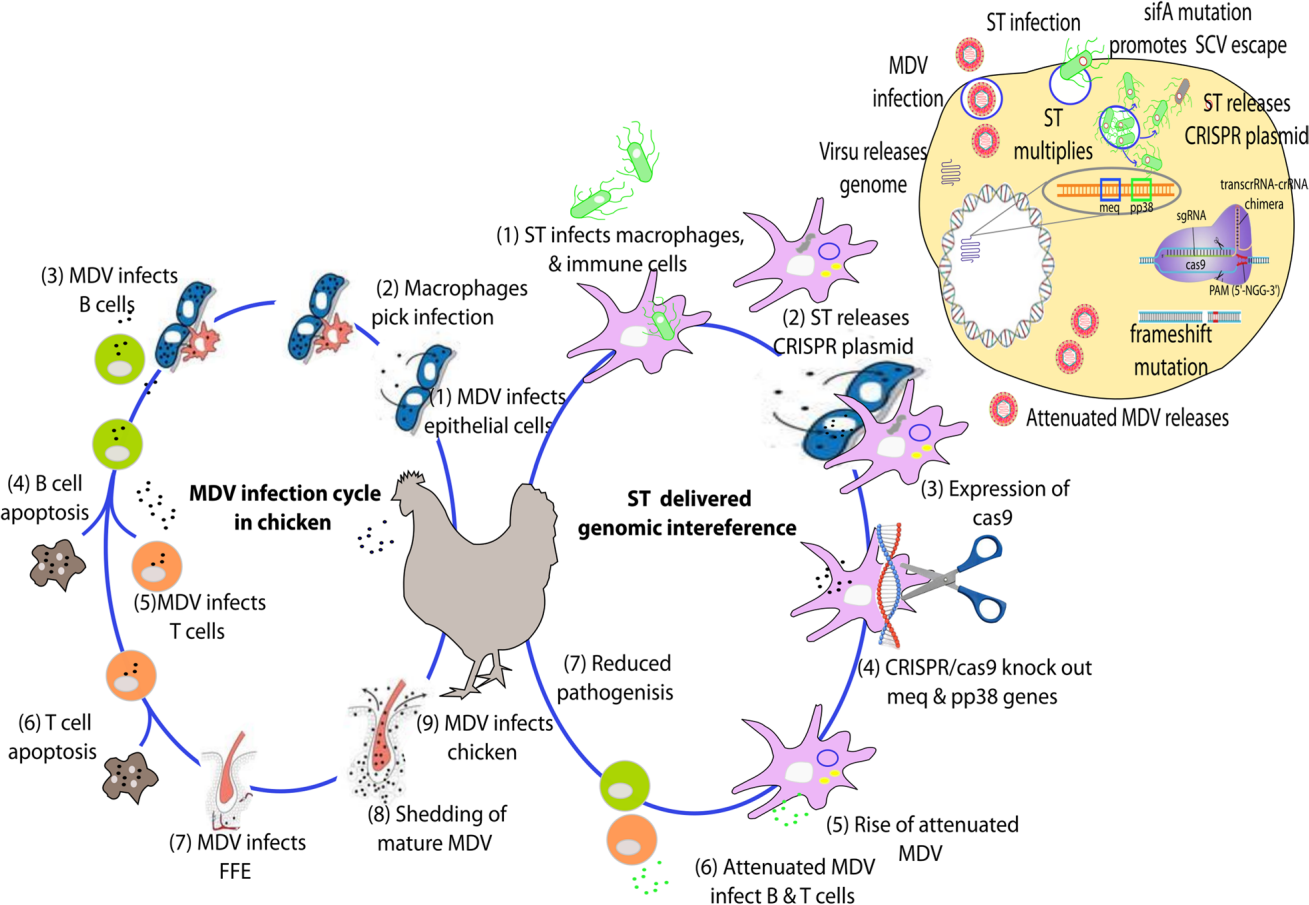
**Proposed mechanism of action of the *Salmonella-*delivered CRISPR/Cas9 system for the specific knockout of the MDV genes *meq* and *ovo* in experimental chickens.** MDV enters chickens mostly via the respiratory epithelium and continues to infect macrophages, B cells, T cells, and other peripheral white blood cells. MDV particles circulate throughout the body, finally reaching feather follicles where they mature and are shed. To knock out the invading MDV genome, attenuated *Salmonella* delivers the CRISPR/Cas9 system to macrophages and tissues such as the lymph nodes, spleen, and liver, where cells are invaded and undergo random lysis by releasing the CRISPR/Cas9 plasmid. The expression of Cas9 may knock out the *meq* and *pp38* genes, severely attenuating MDV. This may lead to reductions in viral activity and the ability to transform healthy tissues into cancerous ones.

The CRISPR/Cas9 system is a form of natural molecular immunity found in bacterial and archaeal species that enables these prokaryotes to defend against viral infections [36]. This remarkable ability has been exploited in CRISPR/Cas9 genome editing platforms, which have shown enormous therapeutic potential for combatting various diseases, such as viral infection, and use as cancer treatments and gene-corrective medicine. Most viral infections in humans and animals that lack vaccination strategies do not have effective preventive medicine, as such treatments intend to alleviate symptoms and leave host immunity to fight against the infection. Studies with CRISPR/Cas9 have demonstrated successful outcomes against hepatitis B virus in vitro conditions [37] and in treating latently infected HIV patients. Nevertheless, the major impediment in the translation of the CRISPR/Cas9 system to in vivo conditions is the lack of efficient delivery systems. As a model study, we constructed an ST-mediated CRISPR/Cas9 delivery system to exert genomic interference against highly virulent Marek’s disease virus in chickens (Figure 8). As chickens are highly susceptible to MDV and the symptoms are very obvious, the treatment outcome could be assessed based on observable symptoms both outside and in internal organs by assessing pathological features. The rationale underlying the present study is supported by the complete abrogation of MDV infection under in vitro conditions when multiple genes are targeted by a CRISPR/Cas9 platform [20]. The principal objective of the present study was not to create a universal vaccine against MDV in chickens but to evaluate whether *Salmonella* can be a useful tool for future CRISPR/Cas9-mediated genomic interference against diseases, such as viral infections and cancer.

To construct an efficient, effective, and safe *Salmonella* delivery system, the strain must retain virulence but be attenuated in a perfect balance. To turn *Salmonella* into an efficient plasmid delivery vector, two genes were deleted from its genome, namely, *lon* and *sifA*. The protease Lon is an attenuation marker that creates *Salmonella* hyperinvasiveness but is unable to cause persistent infection due to impaired cell proliferation. Deletion of the *sifA* gene specifically enables ST to evade the *Salmonella*-containing vacuole (SCV) in host cell cytoplasm by preventing maturation of the SCV into *Salmonella*-containing filaments [38]. This may positively affect intracellular plasmid delivery efficacy by enhancing the number of bacteria in the cytoplasm. Once a chicken was treated with the ST-mediated CRISPR/Cas9 system, it was challenged with a virulent MDV strain. Then, to quantitatively evaluate the viral burden in the chicken, a previously described qRT–PCR method utilizing the virus-encoded *meq* gene as an indicator gene and the *ovo* gene as an internal housekeeping gene was adapted for the animal experiment [29].

During natural infection, viral particles are taken up by the respiratory epithelium (Figures 1B and 8). Then, the viral particles enter circulating immune cells, such as macrophages, and progress into feather follicles, where they mature and continuously shed into the environment. Viral particles could be detected in the spleen, liver, and peripheral blood mononuclear cells of infected animals as early as 7 days and were stably present for the whole lifespan. To evaluate the timing-dependent effect of CRISPR/Cas9 plasmid delivery into the spleen, liver, and other lymphatic tissues during the early stage of viral infection, two test groups that varied in infection timing were created: one group was infected with the virus first (Group C), and the other group was infected with ST harbouring the CRISPR/Cas9 constructs first (Group D). Five days later, we switched the infections in these groups by infecting Group C with ST harbouring CRISPR and Group D with viral particles. Booster ST treatment was conducted on the 7^th^ day for Group C and D birds to maintain infection with the ST strain harbouring the CRISPR/Cas9 plasmid construct. Both MDV and ST target the host lymphoid system during the infection process. Therefore, to achieve CRISPR/Cas9-mediated genomic interference, *Salmonella* would be an ideal choice because it can directly deliver plasmids into antigen-presenting cells, such as macrophages, and tissues in the lymphoid system.

Among the treatment groups, significant resistance against MDV infection was observed in Group D chickens, which were infected with ST before infection with MDV. The maximum ST-mediated plasmid delivery efficacy in splenocytes was observed between 3 and 7 days post ST infection. Therefore, plasmid delivery coincided with early viral infection in Group D animals; thus, the CRISPR system could knock out the target *pp38* gene in the genome of arriving viral particles. This effect might cause a reduction in early numbers of viral particles and may also promote attenuated viral progeny with impaired virulence. This phenomenon possibly generates much attenuated viral progeny, which might be the reason underlying the significant resistance demonstrated by Group D animals, which were first treated with ST harbouring the CRISPR/Cas9 system. This is evident from the qRT–PCR Ct values for Group D animals, which were significantly higher than those for the other test groups as early as 7 days post-viral infection. The difference in Ct values obtained by qRT–PCR showed an increasing trend in Group D animals, with the maximum difference occurring at 28 days post-viral infection. This observation also coincided with a reduced viral copy number, minimal effect on body weight reduction, and significantly extended time to complete paralysis in Group D animals compared to animals in other groups. The second highest efficacy was shown in Group C, in which viral infection occurred before ST treatment. The control groups, Groups B (PBS control) and E (ST vector control), seemed to be severely affected by MDV infection, as most test animals became completely paralyzed and died as early as 6 weeks post-viral infection. We also noted that the genomic interference exerted by targeting a single virulence target was insufficient for the complete abrogation of the disease caused by MDV. Additionally, due to the high virulence of the MDV challenge strain, none of the chickens completely recovered from infection; however, an extended lifespan and reduced pathogenicity were evident. This study suggests that *Salmonella* can be used as an effective and efficient plasmid delivery vector in CRISPR/Cas9 therapy. The expression of the *pp38* gene of MDV has been reported to increase the activity of the bidirectional promoter between the *pp38* gene and 1.8 kb mRNA [23]; hence, possible knockout of *pp38* activity may significantly attenuate viral activity [24, 39]. Studies on *pp38* highlight that the encoded protein is essential for early cytolytic infection in lymphocytes [40]. Thus, knocking out pp38 activity may cause significant attenuation of affected virions. Both of these actions may occur in CRISPR-treated chickens, which could be demonstrated by a significant reduction in virus-induced lesions in the spleen of ST-treated animals compared to that of control animals.

The use of attenuated ST strains in CRISPR/Cas9-mediated therapy could be a safe alternative to the use of viral models and other strategies, such as cancer-derived exosomes. In addition, the thoroughly investigated genome of ST enables easy manipulation of this pathogen for customized delivery strategies. The therapeutic outcome of the ST-delivered CRISPR/Cas9 system for specific genomic interference targeting the virulence-related gene *pp38* demonstrates the breadth and applicability of *Salmonella*-mediated therapy for in vivo applications. Further development may be necessary to enhance the plasmid delivery efficacy of the ST strain, such as programmed lysis strategies suitable for in vivo CRISPR/Cas9 therapy and targeting multiple gene targets for significant disease prevention, to develop safe and effective *Salmonella-*mediated CRISPR therapies for human and animal diseases in the future.

## Supplementary Information

**Additional file 1. The reference *pp38 *gene of *Gallid herpesvirus* 2.** The reference sequence of the *pp38 *gene of *Gallid herpesvirus* 2 NC_002229.3:c127787-126421 is shown. The green highlighted region demarcates the sgRNA sequence. The magenta highlighted region is the PAM sequence.

**Additional file 2. The serum cytopathic effect of Marek’s disease virus on infected chicken fibroblasts.** Serum collected from chickens on the 5^th^ week post-challenge was used to treat cultured chicken fibroblasts at a 1:50 dilution. The cytopathic effect was observed after three days of infection.

**Additional file 3. Symptom severity in control and CRISPR/Cas9-treated chickens**. Chickens (*n* = 2) were sacrificed on the 6^th^ week post-infection. Organs were aseptically harvested. Prominent signs of infection were obvious in spleen and liver specimens. Arrows demarcate MDV-induced symptoms resembling lesions. Spleens from PBS-treated and vector-only control animals showed severe damage caused by MDV due to cancerous transformation. The other two groups, the MDV-1^st^ and ST-1^st^ group, showed a significant delay in the emergence of lesions, indicating resistance against MDV infection.

## Data Availability

The raw and processed data required to reproduce these findings are available from the corresponding author upon request.

## Abbreviations

MDV: Marek’s disease virus
CRISPR/Cas9: clustered regularly interspaced short palindromic repeats/Cas9
ST: *Salmonella* Typhimurium
GFP: green fluorescent protein
