# Supplementary material for: Genetic interference exerted by Salmonella-delivered CRISPR/Cas9 significantly reduces the pathological burden caused by Marek’s disease virus in chickens
Source: Vet Res. 2021 Sep 30;52:125. doi: 10.1186/s13567-021-00995-x (PMC8482593; doi:10.1186/s13567-021-00995-x)
Supplement: Supplementary file 1 — Additional file 1. The reference pp38 gene of Gallid herpesvirus 2. The reference sequence of the pp38 gene of Gallid herpesvirus 2 NC_002229.3:c127787-126421 is shown. The green highlighted region demarcates the sgRNA sequence. The magenta highlighted region is the PAM sequence. [file 13567_2021_995_MOESM1_ESM.docx]

**Additional file 1.**

>NC_002229.3:c127787-126421 Gallid herpesvirus 2, complete genome-pp38 Sequence

ATGGAATTCGAAGCAGAACACGAAGGGCTGACGGCGTCTTGGGTCGCCCCCGCTCCCCAGGGTGGAAAAG

GGGCGGAGGGCCGCGCAGGGGTCGCCGACGAGGCAGGGCATGGGAAAACAGAAGCGGAATGCGCCGAGGACGGCGAGAAATGCGGGGACGCCGAGATGAGCGCTTTGGATCGGGTCCAGAGGGACCGGTGGAGATTCAGTTCTCCGCCCCCTCACTCTGGAGTCACGGGGAAGGGGGCTATTCCAATAAAGGGTGATGGGAAGGCGATAG

AATGCCAGGAGCTAACCGGAGAGGGAGAGTGGCTGTCACAGTGGGAGGAGCTACCGCCTGAGCCCCGGAG

GTCAGGGAATGAACATCTTGACGAAAGTCGGTATGCGAAACAAACCGAAAGGGGTAGCTCTACGGGGAAAGAAGAGGGAGATGGTATGAAGCAGATGGGGGAGCTTGCCCAGCAGTGCGAAGGAGGAACATATGCGGACTTGCTTGTCGAAGCAGAGCAAGCTGTTGTACATTCCGTTCGCGCATTAATGCTGGCCGAAAGACAAAACCCAAATATATTGGGGGAGCATTTGAATAAAAAACGGGTTCTTGTACAACGACCCCGTACTATTCTATCCGTGGAGTCAGAGAATGCAACAATGCGTTCTTATATGCTGGTTACATTGATCTGTTCTGCAAAATCATTATTACTAGGATCGTGCATGTCATTTTTCGCTGGTATGTTAGTCGGTAGAACGGCAGACGTAAAAACACCATTATGGGATACTGTATGTTTGTTAATGGCTTTCTGTGCAGGCATTGTCGTTGGGGGAGTGGATTCTGGGGAGGTGGAATCTGGAGAAACAAAATCTGAATCAAATTAAATTTAATACAGTGTAGCCGTACCCGACGTTGGAGGCGGAGATTAAGCGAATTCTCACCTTTACGAATATTGGTGCAGACAAAGACCAAAAAATGGAAAATGGACAGCTGCAGCACGAAAGTCTCGATTTGGATGCAGATGCCGTTTCTATACCCGAGACTATCTCCCCACCAATCGAGGAAGAACCTGTGCTTTCAGATATTGATGAACAATCAGAATATATTCATTTACAATTAGAATCGGTTACCAGATACAATAATTCCGCACTGTTGCCCACATACGATGATGCAGTTGACCCACCCCCTTCATACGATTCCCTATCCCCGATACATAATGTTAACAATTCTGAAAGTTGCGCAGAAGTTGACTTGCGTTTTATCATTCGACATGATGGATGTGCGATCGCTACATTATTAATACTTTTTTTGACGGTAGTTTCTGCAACCCTTGTAACTATTATCACAGAAACATAATTGACGTATGTGATACAATAAA
