# Supplementary figures and images for: Genetic interference exerted by Salmonella-delivered CRISPR/Cas9 significantly reduces the pathological burden caused by Marek’s disease virus in chickens
Source: Vet Res. 2021 Sep 30;52:125. doi: 10.1186/s13567-021-00995-x (PMC8482593; doi:10.1186/s13567-021-00995-x)

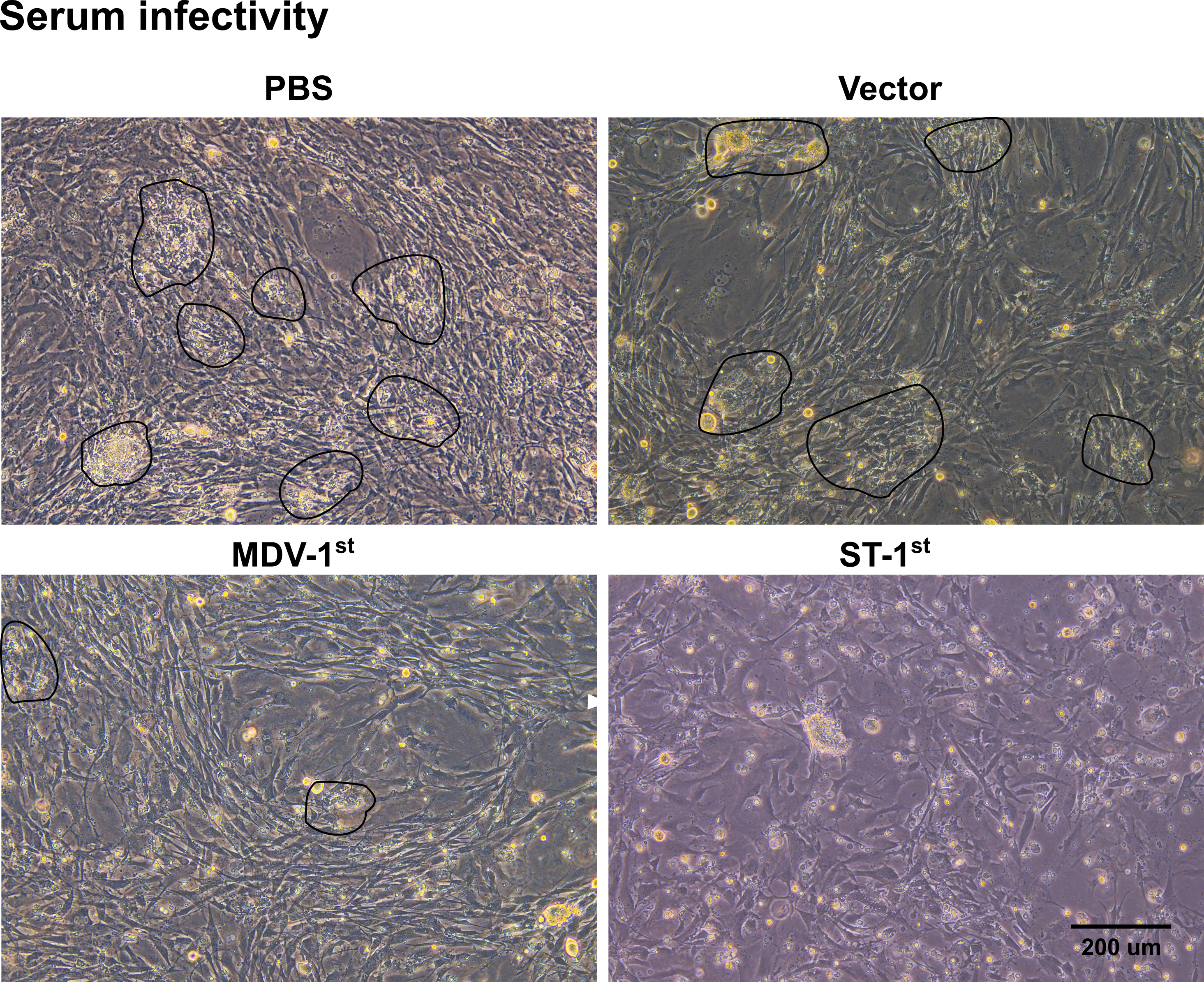

Supplement: Supplementary file 2 — Additional file 2. The serum cytopathic effect of Marek’s disease virus on infected chicken fibroblasts. Serum collected from chickens on the 5th week post-challenge was used to treat cultured chicken fibroblasts at a 1:50 dilution. The cytopathic effect was observed after three days of infection. [file 13567_2021_995_MOESM2_ESM.tif]

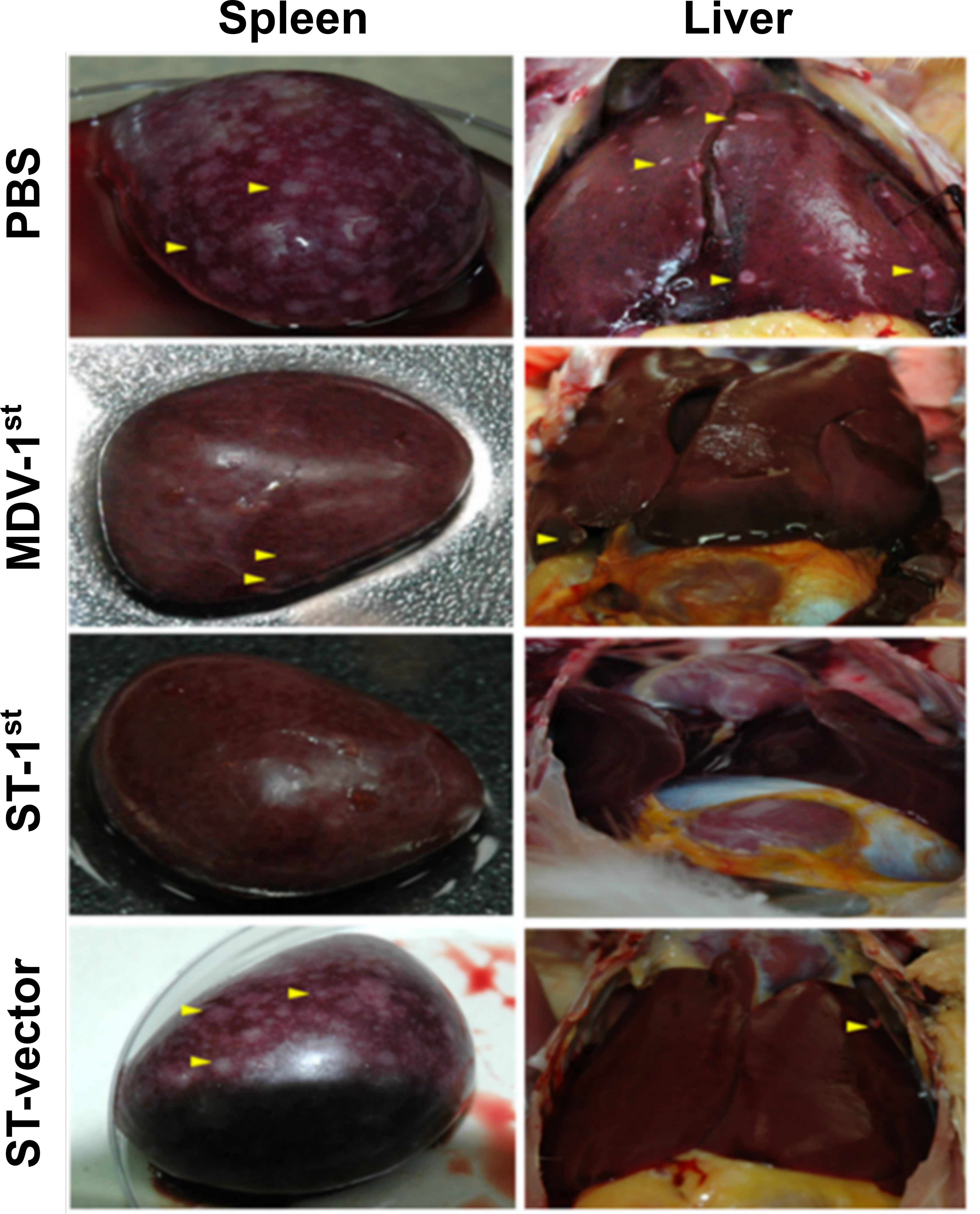

Supplement: Supplementary file 3 — Additional file 3. Symptom severity in control and CRISPR/Cas9-treated chickens. Chickens (n = 2) were sacrificed on the 6th week post-infection. Organs were aseptically harvested. Prominent signs of infection were obvious in spleen and liver specimens. Arrows demarcate MDV-induced symptoms resembling lesions. Spleens from PBS-treated and vector-only control animals showed severe damage caused by MDV due to cancerous transformation. The other two groups, the MDV-1st and ST-1st group, showed a significant delay in the emergence of lesions, indicating resistance against MDV infection. [file 13567_2021_995_MOESM3_ESM.tif]
